# Royal Jelly and *trans*-10-Hydroxy-2-Decenoic Acid Inhibit Migration and Invasion of Colorectal Carcinoma Cells

**DOI:** 10.17113/ftb.60.02.22.7495

**Published:** 2022-06

**Authors:** Milena M. Jovanović, Dragana S. Šeklić, Jelena D. Rakobradović, Nevena S. Planojević, Nenad L. Vuković, Milena D. Vukić, Snežana D. Marković

**Affiliations:** 1 Department of Biology and Ecology, Faculty of Science, University of Kragujevac, Radoja Domanovića 12, 34000 Kragujevac, Serbia; 2 Institute for Information Technologies, Department of Natural Sciences, University of Kragujevac, Jovana Cvijića bb, 34000 Kragujevac, Serbia; 3 Institute for Oncology and Radiology of Serbia, Pasterova 14, 11000 Belgrade, Serbia; 4 Department for Chemistry, Faculty of Science, University of Kragujevac, Radoja Domanovića 12, 34000 Kragujevac, Serbia

**Keywords:** colorectal cancer, epithelial-mesenchymal transition (EMT), dietary supplement, migration of carcinoma cells, Snail, *trans*-10-hydroxy-2-decenoic acid

## Abstract

**Research background:**

Acquisition of migratory potential is pivotal for cancer cells, enabling invasion and metastasis of colorectal carcinoma. Royal jelly and its bioactive component *trans*-10-hydroxy-2-decenoic acid (10H2DA) showed remarkable antimetastatic potential, but the molecular mechanism underlying this activity is unclear.

**Experimental approach:**

Identification and quantification of 10H2DA in royal jelly originating from Serbia was done by HPLC method. Cytotoxicity of 10H2DA was measured by tetrazolium dye MTT test in concentration range 1-500 μg/mL after 24 and 72 h. Its effect on the collective and single-cell migration was measured by wound healing and transwell migration assays. Invasive potential of cancer cells was evaluated by a transwell method modified with collagen. Immunofluorescence was used for migratory and invasive protein expression, while the gene expression of these markers was evaluated by quantitative real time polymerase chain reaction (qRT-PCR). All assays were applied on human colorectal carcinoma HCT-116 and SW-480 cell lines and, except for MTT, evaluated after 24 h of treatment with two selected concentrations of royal jelly and 10H2DA.

**Results and conclusions:**

According to HPLC, the mass fraction of 10H2DA in royal jelly was 0.92% (*m*/*m*). Treatment with 10H2DA showed no cytotoxic effect; however, significant inhibitory potential of royal jelly and 10H2DA on the motility and invasiveness of colorectal cancer cells was observed. More pronounced effect was exerted by 10H2DA, which significantly suppressed collective cell migration and invasiveness of SW-480 cells, as well as single- and collective cell migration and invasive potential of HCT-116 cell line. Treatments increased epithelial markers E-cadherin and cytoplasmic β-catenin in HCT-116 cells, thus stabilizing intercellular connections. In SW-480 cells, 10H2DA increased E-cadherin on protein and gene level, and suppressed epithelial-mesenchymal transition (EMT) markers. In both cell lines, treatments induced significant suppression of promigratory/proinvasive markers: N-cadherin, vimentin and Snail on protein and gene level, which explains decreased migratory and invasive potential of HCT-116 and SW-480 cells.

**Novelty and scientific contribution:**

Our study presents new findings and elucidation of royal jelly and 10H2DA molecular mechanism that underlies their antimigratory/antiinvasive activity on colorectal cancer cells. These findings are shown for the first time indicating that these natural products are a valuable source of anticancer potential and should be reconsidered for further antitumour therapy.

## INTRODUCTION

Colorectal cancer (CRC) is the third most common type of cancer, responsible for numerous deaths worldwide (*1*). Metastasis in malignant tumours causes a lethal outcome and newly diagnosed cases are usually at advanced stages, with metastases already present in the body (*2*).

Motility is an essential property of cancer cells; their dissemination implies migration/invasion through extracellular matrix and is of particular interest for cancer research (*3*). As previously revealed, cancer cells can migrate utilizing two strategies: in a collective way and in the form of single cells (*3*). Collective migration implies coupling between neighbouring cells, taking collective decisions, preserving stability of intercellular junctions, and moving as a coherent entity (*3*). Epithelial-mesenchymal transition (EMT) and inhibition of Wnt/β-catenin signalling pathway contribute to migratory potential of cancer cells, alongside with overexpression of promigratory/invasive proteins: N-cadherin, Snail, vimentin, and β-catenin (*4*, *5*). Formation of adherent junctions between neighbouring cells implies coupling of the epithelial marker E-cadherin and the intercellular protein β-catenin (*6*). During metastasis, these junctions are usually dysregulated in cancer cells, where β-catenin becomes localized in the nuclei of the cells that form an invasive front (*7*). Snail, as an important EMT marker and transcription factor, induces expression of migratory/invasive markers (like N-cadherin and vimentin), and is activated by repression of E-cadherin during EMT (*6*-*8*). N-cadherin affects cell adhesion and is upregulated in cancer cells inducing downregulation of E-cadherin (*i.e.* cadherin switch) (*6*, *9*). Vimentin is the marker of the mesenchymal cell phenotype and is expressed during metastasis, stimulating migration/invasiveness of CRC cells and contributing to successful collective migration (*5*, *6*).

Besides abnormal expression of the aforementioned markers, nutrition is also responsible for increased risk of carcinogenesis (*1*). However, certain natural products prove to be effective in anticancer treatment (*10*). Royal jelly has been traditionally used as a dietary supplement with several pharmaceutical and significant anticancer properties (*11*, *12*), which is the reason why we tested it on colorectal carcinoma cells. One of royal jelly crucial components, unsaturated fatty *trans*-10-hydroxy-2-decenoic acid (10H2DA), not found in any other natural product, proved to affect the invasion of cancer cells *in vivo* and *in vitro* (*12*, *13*). However, the molecular mechanism of this 10H2DA activity is still unknown concerning CRC and its effects on Wnt/β-catenin pathway and promigratory proteins.

The aim of this research is assessment of 10H2DA content in royal jelly, cytotoxicity and antimigratory/invasive potential of royal jelly originating from Serbia and commercially available 10H2DA on two colorectal cancer cell lines (HCT-116 and SW-480). As far as we know, this study analyses molecular mechanisms of migration/invasion of colorectal carcinoma cells treated by these products for the first time.

## MATERIALS AND METHODS

### Materials

HPLC grade methanol and acetic acid were from J.T. Baker (Deventer, The Netherlands). Dulbecco’s modified Eagle medium (DMEM) was obtained from Lonza, Basel, Switzerland. Phosphate-buffered saline (PBS), penicillin and streptomycin were from Gibco, Invitrogen, Thermo Fisher Scientific (New York, NY, USA). Foetal bovine serum (FBS) was from Capricorn Scientific (Ebsdorfergrund, Germany). Collagen type I (rat tail) was purchased from Corning (New York, NY, USA), while 3-(4,5-dimethylthiazol-2-yl)-2,5-diphenyltetrazolium bromide (MTT), dimethyl sulfoxide (DMSO), Triton X-100, bovine serum albumin, crystal violet, 2-(N-morpholino)ethanesulfonic acid (MES), chloroform and isopropanol were from SERVA (Heidelberg, Germany). TRIzol and molecular biology grade water were from Ambion (Austin, TX, USA). cDNA reverse transcription and qPCR kit were from EURx (Gdańsk, Poland). Primers for *E-cadherin, β-catenin* and *N-cadherin* gene expression were from Mycrosynth (Balgach, Switzerland), while primers for *vimentin* and *Snail* were synthesized by Metabion (Planegg, Germany). Paraformaldehyde was from Merck (Darmstadt, Germany). Polyvinyl alcohol mounting medium was from Fluka Analytical (Buchs, Switzerland). β-catenin primary antibodies were from Invitrogen Corporation (Camarillo, CA, USA). N-cadherin and Snail primary antibodies were from Abcam (Cambridge, UK). E-cadherin primary antibody was obtained from BD Systems (Bury St Edmunds, UK). Vimentin primary antibody was from Dako (Glostrup, Denmark). Secondary antibodies conjugated with Cy3, Аlexa Fluor 448 and DAPI (diamidino-2-phenylindole) were from Thermo Fisher Scientific (Waltham, MA, USA).

### Royal jelly and 10-hydroxy-trans-2-decenoic acid

Fresh royal jelly sample (*Apis mellifera* L.) was obtained from beekeepers located in Velika Plana, Podunavlje District in Central Serbia (44°20’02”N, 21°04’36”E) in 2017. The sample was kept frozen at -20 °C and protected from light from the time of obtaining until the analysis. Royal jelly acid, 10H2DA, was purchased from TCI Chemicals (Tokyo, Japan).

### Chemical characterization

The royal jelly sample (30 mg) was homogenized, weighed on analytical balance, dissolved in mobile phase (*φ*(methanol, water)=45% and *φ*(acetic acid, water)=1%), vortexed for 15 min at 3000×*g* and filtered (0.22 μm membrane filter; Sigma Chemicals Co., Merck, St Louis, MO, USA). A Shimadzu Prominence HPLC system (Duisburg, Germany) consisting of LC-20AT pump, DGU-20A degasser, CTO-20A column oven, 20 μL loop, SPD-M20A photodiode-array (PDA) detector and CBM-20A Prominence communication module was used. A Thermo Hypersil GOLD aQ C18 column (150 mm×4.6 mm, 5 μm; Thermo Fisher Scientific, New York) was employed. The isocratic mode of elution was performed using a mobile phase consisting of *φ*(methanol, water)=45% and *φ*(acetic acid, water)=1%. The flow rate was 0.5 mL/min, the injection volume (loop) was 20 μL, while the column oven was adjusted to 35 °C. Total run time was 35 min. The monitoring wavelength for quantification of 10H2DA was 225 nm. UV/Vis spectra were monitored between 200 and 400 nm.

Stock solution was prepared by dissolving 1 mg 10H2DA in methanol (1 mL), while for calibration curve, standard solutions with concentrations of 50, 25, 12.5, 6.25, 3.125 and 1.56 μg/mL were used. The experiment was repeated 3 times, and the concentration of investigated compound was determined from the peak areas, using the equation for linear regression obtained from the calibration curve (R^2^=0.9989). The limits of detection (LOD) and quantification (LOQ) for 10H2DA were determined under the above-described chromatographic conditions at signal-to-noise ratios of approx. 3 and 10, while the ranges of linearity were determined from the calibration curves (plotting peak area *versus* concentration; three injections).

### Cell line analyses

Immortalized adherent human colorectal carcinoma cell lines HCT-116 and SW-480 were obtained from American Tissue Culture Collection (ATCC, Manassas, VA, USA), and cultured in DMEM with 10% (*V*/*V*) FBS, 100 U/mL penicillin and 100 μg/mL streptomycin in humidified atmosphere (5% CO_2_, 37 °C). Cells were grown in 75 cm^2^ surface area cell culture flask until 70-80% confluence was reached, after which the cells were seeded for different assays.

Royal jelly was dissolved in PBS, diluted with DMEM, obtaining a stock solution (1 mg/mL) further diluted with culture medium to various concentrations. The 10H2DA was dissolved in DMSO and DMEM, obtaining 100 mM stock solution and diluted with medium to working concentrations.

### Cell viability assay

The effects of 10H2DA (0.1, 1, 10, 50, 100 and 500 µM) on HCT-116 and SW-480 cell viability were evaluated within 24 and 72 h using MTT assay (*14*). The effects of royal jelly on these colorectal cancer cell lines were assessed in our previous study (*15*). The cell viability was calculated as a ratio of the absorbance of the treated cells divided by the absorbance of the control (untreated cells), multiplied by 100 to give percentage viability. The IC_50_ values (concentration that corresponds to a death rate of 50% of cells) were calculated based on cytotoxic values obtained by the MTT assay and generated as a plot of cytotoxicity (in %) *versus* sample concentrations.

### Migration assays

**Fig. S1:**
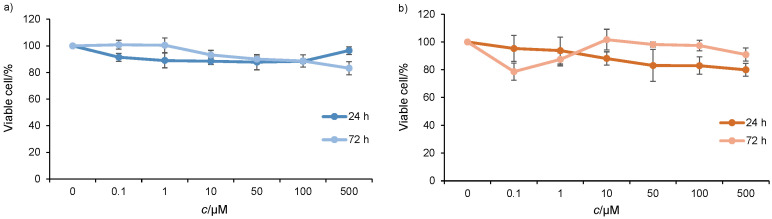
Effects of *trans*-10-hydroxy-2-decenoic acid (10H2DA) on: a) HCT-116 and b) SW-480 cell viability assessed with MTT assay

Collective cell migration was analysed by wound healing (scratch) assay (*16*), based on making a scratch in a confluent cell layer and monitoring the behaviour and movement of cells over time. The cells at the edge of the wound began to move, filling and closing the wound. HCT-116 and SW-480 cells were seeded into 12-well plates (7·10^5^ cells per well; Thermo Fisher Scientific, New York) and within 24 h, when 90-100% confluence was reached, growth medium was aspirated, and a scratch in confluent cell monolayer was made using a sterile plastic disposable pipette tip (10 µL; Thermo Fisher Scientific, New York). The cells were then washed twice with PBS to remove detached cells and treated with 1 mL of investigated substances at sublethal concentrations (royal jelly: 10 and 100 µg/mL; 10H2DA: 10 and 100 µM). The cytotoxicity of 10H2DA was determined by MTT assay (Fig. S1) following the procedure used in our previous work on royal jelly (*15*). Migration of cells was monitored with an inverted NICON Eclipse-Ti microscope (Nikon Instruments Inc., Melville, NY, USA) and micrographs were taken at 100× magnification after 0, 12 and 24 h of treatment. The healing rate was quantified by measuring the distance between cells at the edges of the wound by using ImageJ software package (*17*) and calculated as a ratio of the values of the treated group divided by the values of the control group (untreated cells), multiplied by 100 to give percentage of relative wound space. The degree of cell migration was determined by comparing images from the moment of inflicting the wound (0 h) up to the expiration of a certain period (12 and 24 h), and the results are presented as relative wound space (in %).

Impact of treatments on single HCT-116 and SW-480 cell migration was assessed by transwell method (*18*), where 10^5^ cells were seeded in the upper chambers of inserts (8 µm pore size; Greiner Bio-One, Leipzig, Germany) and treated with 10 and 100 µg/mL royal jelly, or 10 and 100 µM 10H2DA, while 20% FBS was used as attractant in lower chambers. Migratory potential of cells was analysed after 24 h of treatment, while the untreated cells served as control. The cells were fixated using 4% formaldehyde, followed by the removal of non-migrated cells from the top of each insert with a cotton swab. Then, they were stained with 0.1% crystal violet in MES buffer for 10 min and the membranes were destained in 10% acetic acid. The absorbance was measured at 595 nm on the ELISA reader (RT-6100; Rayto Life and Analytical Sciences Co., Hamburg, Germany), and the obtained values that represent cell migratory potential are expressed as the index of migration/absorbance (*A*).

### Invasion assay

The capacity of cancer cells to invade is characterized by their movement through a three-dimensional matrix. Thus cancer cells are remodelling the matrix, which enables the aggressive behaviour of solid cancers. We assessed the invasive potential of control (untreated) and treated HCT-116 and SW-480 cells by applying transwell technique with modifications, and the assay was adapted from the previously described work (*19*). The upper side of the inserts was overlaid with a thin collagen layer (150 µL) mixed with DMEM, PBS and NaOH, and left at 37 °C in a humified atmosphere of 5% CO_2_ for at least 30 min to jellify. On top of collagen, 10^5^ cells were plated, treated with 10 and 100 µg/mL royal jelly, or 10 and 100 µM 10H2DA, and allowed to invade for 24 h. Invasive cells degraded the matrix and moved through the collagen layer, whereas the non-invasive cells were blocked from migrating through the collagen. We measured the invasiveness of the attached cells on the underside of the porous membrane by staining with 0.1% crystal violet in MES buffer for 10 min. Destaining of membranes was done in 10% acetic acid, after which the absorbance was measured at 595 nm on the ELISA reader (RT-6100; Rayto Life and Analytical Sciences Co.). The obtained values that represent cell invasive potential are expressed as index of invasion/absorbance (*A*).

### Immunofluorescent staining

The E-cadherin, cytoplasmic and nuclear β-catenin, N-cadherin, vimentin and Snail protein expression was evaluated using immunofluorescent staining according to Šeklić *et al.* (*18*). HCT-116 and SW-480 cells were plated at 40 000 cells/well on glass coverslips (Thermo Fisher Scientific, New York) in 6-well plates and treated with royal jelly (10 and 100 µg/mL) or 10H2DA (10 and 100 µM). Immunofluorescent staining was performed 24 h after treatment. Cells were washed with PBS and fixed with 4% *p*-formaldehyde for 20 min at 37 °C. After washing with PBS, 0.1% Triton-X was used for permeabilization of cells in order to detect vimentin, while for detection of nuclear and cytoplasmic β-catenin, the cells were permeabilized with 0.25% Triton-X. Protein expression of N-cadherin, E-cadherin and Snail was evaluated after permeabilization of cells in cold methanol. Non-specific binding sites were blocked with 1% BSA for 20 min. Sample coverslips were stained with specific monoclonal primary antibodies (diluted in PBS according to manufacturer’s propositions) for 1 h at room temperature and then with secondary antibodies conjugated with Alexa Fluor 488 or Cy3 (1:200 dilution in PBS), along with DAPI dye for nuclear staining (1:1000 dilution in PBS). Inverted fluorescent microscope Eclipse Ti (Nikon Instruments Inc.) was used to obtain micrographs at 600× magnification. Preparation of micrographs for analysis consisted in rendering the colour image into black and white followed by measuring the fluorescence intensity of the target protein in the cells in relation to the background. The fluorescence was quantified as described earlier by Schneider *et al.* (*20*), using the ImageJ software package (*17*), and the results were presented as relative fluorescence per cell (in %).

### Anti- and promigratory gene expression

RNA was isolated according to a protocol described earlier (*15*). Cells were cultured in 25 cm^2^ flasks (Thermo Fisher Scientific, New York), and when confluency over 90% was reached, they were treated with royal jelly (10 and 100 µg/mL) or 10H2DA (10 and 100 µM), while the cells treated with DMEM only served as control. After 24 h, cells were trypsinized and centrifuged (MPW-150R; MPW Med. Instruments, Warsaw, Poland) at 1200×*g* for 10 min, after which they were homogenized in TRIzol and centrifuged (MPW-150R; MPW Med. Instruments) at 12 000×*g* and 4 °C for 5 min. Chloroform was added to each sample, followed by mixing for 15 s, and left to incubate for 2-3 min at room temperature. After centrifugation for 15 min at 12 000×*g* and 4 °C, three visible phases were observed, where the top phase (containing RNA) was transferred to a new microtube. A volume of 500 µL of isopropanol was added, RNA was precipitated for 10 min and samples were centrifuged (12 000×*g* for 10 min at 4 °C). The supernatant was removed, while the precipitate was washed with 1 mL of 70% ethanol, followed by centrifugation at 7500×*g* and 4 °C for 5 min. The supernatant was removed, ethanol was left for air drying (2-3 min), RNA residue was resuspended in 20 µL molecular biology grade water, and then incubated at 55 °C in a Thermomixer® R thermoblock (Eppendorf, Merck, Darmstadt, Germany) for 2-3 min. The concentration of RNA was measured in samples using biophotometer (BioPhotometer Plus, Eppendorf, Hamburg, Germany), where the indicator of RNA purity was considered when ratio of absorbances (*A*_260 nm_/*A*_280 nm_) were in the range 1.8-2.0. Samples were further preserved at -80 °C until use.

Translation of mRNA into cDNA was performed using reverse transcription kit Mastercycler® PCR (Eppendorf, Hamburg). The expression of housekeeping gene *β-actin* and targeted genes: *E-cadherin, N-cadherin, β-catenin, vimentin* and *Snail* (forward and reverse primer sequences are shown in Table S1) was evaluated with a commercially available qPCR kit (EURx). Reaction mixture was made for each target gene separately by adding forward/reverse primer mix (0.5 μL forward primer and 0.5 μL reverse primer, concentration 10 μM) into 10 μL qPCR Master Mix (EURx), followed by the addition of 1 μL cDNA and 8 μL molecular biology grade water. Each qPCR cycle started with polymerase activation step and was repeated in 40 cycles, each of them consisting of three steps: denaturation of DNA at 95 °C for 15 s, primer hybridization (annealing) at 60 °C for 30 s, and extension at 72 °C for 30 s. After amplification using Applied Biosystems 7500 Real-Time PCR (Thermo Fisher Scientific), relative gene expression was calculated in the examined samples according to the formula:

2^-ΔΔCt^=ΔCt1-ΔCt2 /1/

where ΔCt1 is the difference between the cycle threshold (Ct) values of the examined gene in the sample of treated cells and the Ct values of *β-actin* in the sample of treated cells. ΔCt2 is the difference between the Ct values of the examined gene in control cells and Ct values of *β-actin* in control cells.

### Statistical analysis

All the obtained data are expressed as the mean value±standard error (S.E.) and they have normal distribution. Therefore, statistical analysis was done using Student’s *t-*test and one-way ANOVA for multiple comparisons to evaluate statistical significance (p<0.05 was considered as statistically significant). These tests were performed using IBM SPSS Statistics, v. 17 (IBM Corp., New York, NY, USA) for Windows (*21*). The IC_50_ values were calculated using CalcuSyn software (*22*).

## RESULTS AND DISCUSION

### 10H2DA content in royal jelly

**Fig. 1:**
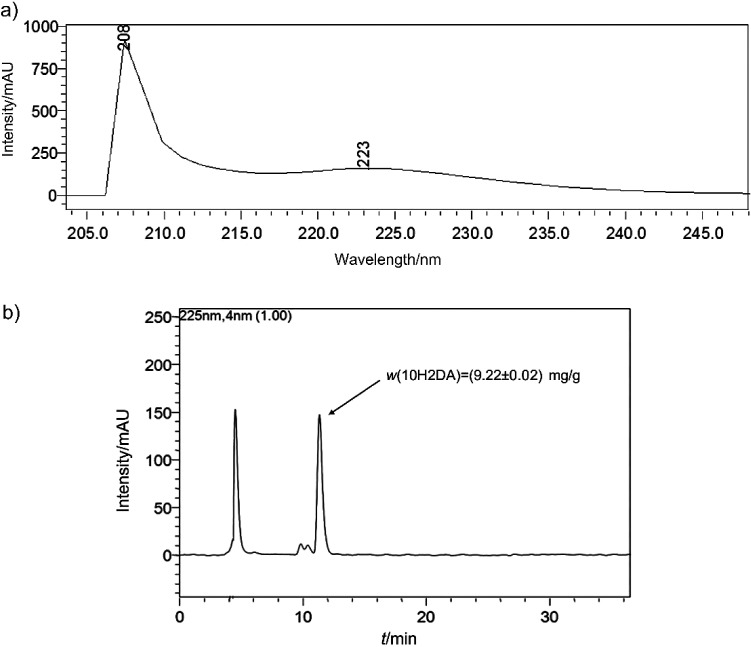
Detection of *trans*-10-hydroxy-2-decenoic acid (10H2DA) in royal jelly sample: a) UV spectra and b) HPLC chromatogram

As a result of the presence of an enone system inside the lipophilic molecule of 10H2DA, the absorption maxima was batochromically shifted above 200 nm (Fig. 1). Thus, an effective, easy and relatively cheap identification and quantification of 10H2DA in royal jelly was obtained using HPLC coupled with photodiode-array detector. The monitoring wavelength for identification and quantification was 225 nm. Under the described isocratic conditions, a chromatographic peak of 10H2DA was successfully separated from two unknown compounds (eluted before) and it appeared at the retention time of 11.31 min. The chromatographic peak of the investigated compound is almost symmetrical (Fig. 1).

Prior to the quantification, it was observed that the concentration of 10H2DA was linear within a concentration range from 1.56 to 50 µg/mL (R^2^=0.9989). The observed limit of detection (LOD) and the limit of quantification (LOQ) were 0.67 and 0.89 µg/mL, respectively. The mass fraction of 10H2DA in the investigated royal jelly sample was (9.22±0.02) mg/g or 0.92% (*m*/*m*).

Literature data from European countries with similar geographic positions reported 10H2DA content in royal jelly in the range 0.8-6.5% (*23*). Our sample obviously contains a mass fraction of 10H2DA that fits within the aforementioned range.

### Cytotoxic activity

Previously, we reported the cytotoxicity of royal jelly on HCT-116 and SW-480 cells (*15*), where royal jelly showed no cytotoxic effect, with IC_50_>500 μg/mL. In this study, 10H2DA also exerted no cytotoxicity on HCT-116 and SW-480 cells (Fig. S1) after both 24 and 72 h, with IC_50_ values higher than 500 μM. The lack of cytotoxicity of royal jelly and 10H2DA on the tested cancer cells is a confirmation of previously reported results on Caco2 and WiDR cell lines (*13*, *24*, *25*).

### Effects on collective and single-cell migration and invasion

Cell migration, invasion and changes in adherent junctions are pivotal steps in cancer metastasis, hence understanding and targeting these processes are crucial in fighting this disease (*3*). The main antitumour therapeutic approaches have not been satisfactory so far, thus attempts to improve treatments have been made with natural products (*13*), such as royal jelly and 10H2DA that have already shown remarkable antimetastatic potential (*12*, *13*). We have assessed for the first time the inhibitory effects of royal jelly and 10H2DA on the type of motility and invasiveness of HCT-116 and SW-480 cells, and more importantly, elucidated possible mechanisms of these actions.

First, these cell lines were established from different pathological stages of colon cancer. Namely, SW-480 were identified as stage II, and HCT-116 cells as stage IV colonic adenocarcinoma (*26*). Hence, HCT-116 cells are poorly differentiated and highly aggressive compared to SW-480 cells, which are characterized as moderately differentiated, more epithelial-like and less mobile (*27*, *28*).

**Fig. 2:**
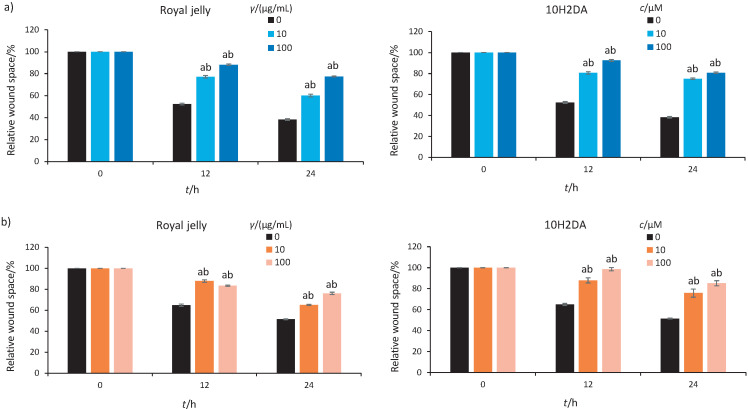
The effects of royal jelly and *trans*-10-hydroxy-2-decenoic acid (10H2DA) on collective migration of: a) HCT-116 and b) SW-480 cells analysed 12 and 24 h after treatment. The analysis of wound space is shown as relative level of changes compared to control cells, presented as percentages. ^a^p<0.05 is considered statistically significant difference between treatment periods compared to 0 h, and ^b^p<0.05 is statistically significant difference between treatment concentrations in a treated group at the same time (*N*=4, two independent experiments). Both royal jelly and 10H2DA suppressed the collective migration of both tested cell lines; however, higher 10H2DA concentration had the most prominent antimigratory effect on both tested cell lines

Cancer cells are able to use different movement strategies, depending on the growth and environmental conditions, and thus can migrate as single cells or in coherent mass (*3*). Collective cell migration implies involvement of cadherins and cell-cell communication. Cytoskeletal activity between neighbouring cells and with the environment is also important in a cohesive cell group (*3*). For assessment and quantification of collective cell migration, we applied wound healing assay, which is suitable for determination of migratory ability of whole cell masses (*3*). The decisive result of our study is that royal jelly and 10H2DA significantly and dose-dependently inhibited the collective migration of both tested cell lines, with 10H2DA showing better effect (Fig. 2 and Table S2). It was observed that 10H2DA had a stronger suppressive effect on migration of HCT-116 cells, especially after 24 h.

Both treatments were able to inhibit collective migration of SW-480 cells statistically and dose-dependently. The 10H2DA showed stronger antimigratory potential against these cells than royal jelly (Fig. 2 and Table S2).

**Fig. 3:**
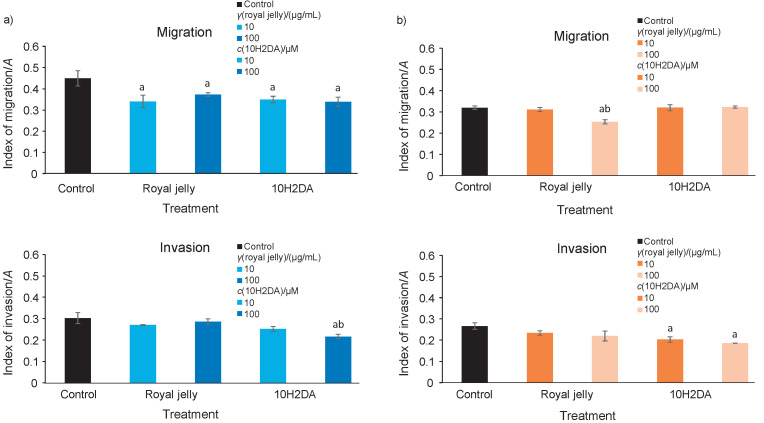
Migratory and invasive potential of: a) single HCT-116 and b) SW-480 cells determined by transwell assay 24 h after royal jelly and *trans*-10-hydroxy-2-decenoic acid (10H2DA) treatment (*N*=4, two independent experiments). ^a^p<0.05 indicates a significant difference compared to control, while ^b^p<0.05 indicates a significant difference between concentrations in a treated group. Both treatments repressed migratory potential of HCT-116 cells, and inhibited invasiveness of both cell lines

Transwell assay is widely used to evaluate migratory behaviour of single cells, in which individual migration implies mesenchymal or amoeboid type of movement that involves activation of matrix metalloproteinases (MMPs) (*3*). According to our results, both royal jelly and 10H2DA were able to significantly inhibit the migratory and invasive potential of single HCT-116 cells, which proved to be more sensitive to treatments than the SW-480 cell line. Furthermore, the effects of 10H2DA were more prominent than of royal jelly, and in dose-dependent manner (Fig. 3 and Table S3).

On the other hand, only higher dose of royal jelly significantly reduced motility of single SW-480 cells, while 10H2DA had no effect (Fig. 3 and Table S3). We can conclude that 10H2DA suppressed collective migration of SW-480 cells, while royal jelly suppressed their single cell migration.

Meanwhile, the treatments suppressed the invasiveness of SW-480 cells significantly and in dose-dependent manner, whereas the strongest suppression was noticed using higher dose of 10H2DA (Fig. 3 and Table S3).

Generally, royal jelly and 10H2DA suppressed collective and single-cell migration of HCT-116 and SW-480 cells, and 10H2DA exerted stronger suppressive activity and was also able to significantly inhibit their invasiveness.

### Evaluation of protein expression

**Fig. 4:**
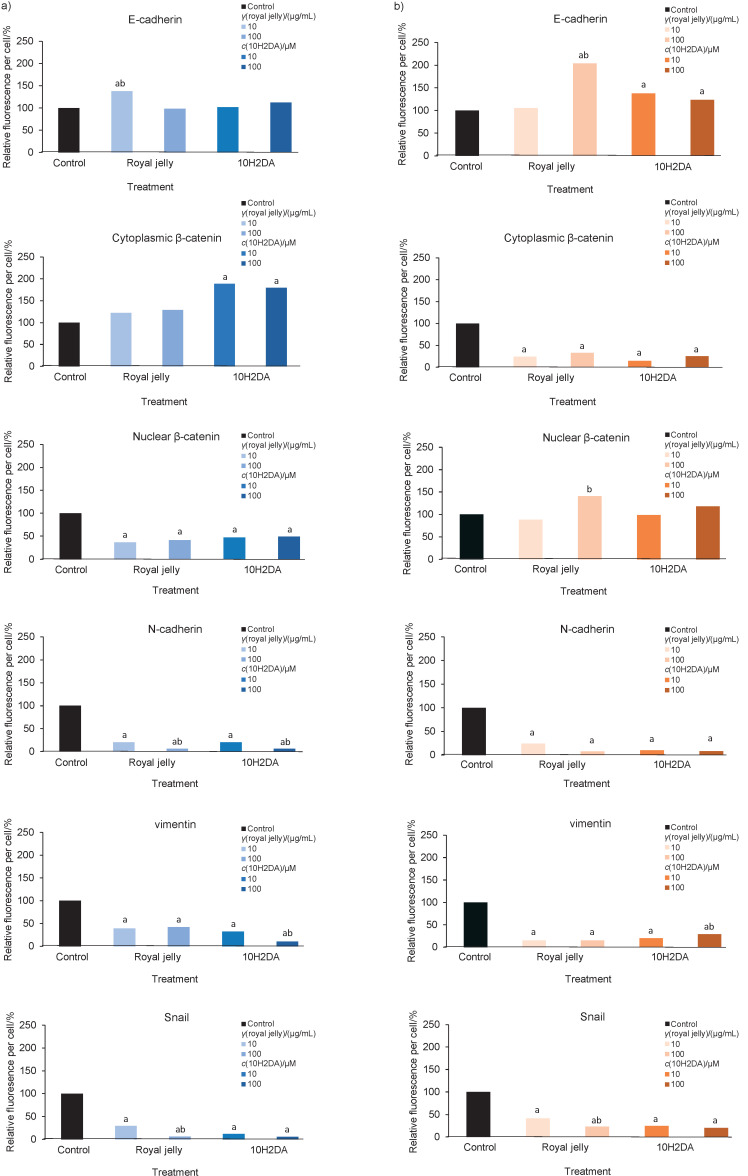
Effects of royal jelly and *trans*-10-hydroxy-2-decenoic acid (10H2DA) on anti- and promigratory/invasive protein expression in: a) HCT-116 and b) SW-480 cells after 24 h, expressed as relative fluorescence per cell (in %) (*N*=2, two independent experiments). Fluorescence was quantified using ImageJ software (*17*); samples with immunolabelled proteins were photographed and at least 30 micrographs were taken per treatment. The procedure was repeated at least four times on each micrograph and at least six cells were analysed per each micrograph. ^a^p<0.05 indicates a significant difference compared to control, while ^b^p<0.05 indicates a significant difference between concentrations in a treated group. Inhibition of promigratory and proinvasive markers by royal jelly and 10H2DA is obvious in both tested cell lines

**Fig. 5:**
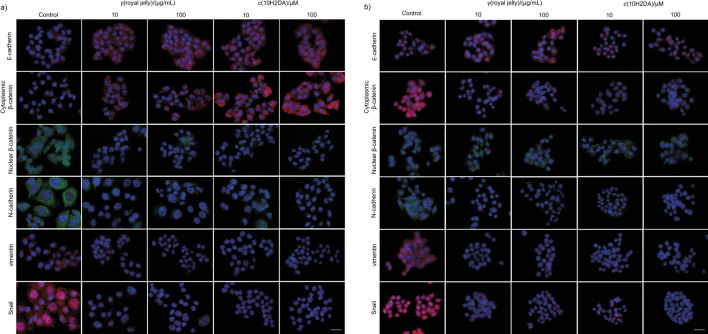
Effects of royal jelly and *trans*-10-hydroxy-2-decenoic acid (10H2DA) on anti/promigratory protein expression in: a) HCT-116 and b) SW-480 cells after 24 h of treatment. Representative micrographs of cells with immunolabelled proteins after treatment with royal jelly (10 and 100 µg/mL) and 10H2DA (10 and 100 µM) captured after 24 h. Scale bar: 50 µm; cell nuclei were stained with DAPI (blue); E-cadherin, cytoplasmic β-catenin, vimentin and Snail proteins were stained with secondary antibody conjugated with Cy3 (red); nuclear β-catenin and N-cadherin proteins were stained with secondary antibody conjugated with Alexa 488 (green). Merged micrographs show suppression of promigratory and proinvasive proteins in HCT-116 and SW-480 cells by both royal jelly and 10H2DA

Next we elucidated the possible mechanism of royal jelly and 10H2DA antimigratory/antiinvasive action. In HCT-116 cells, E-cadherin was increased by all treatments, whereas only a lower dose of royal jelly induced its significant increase. The 10H2DA significantly elevated cytoplasmic β-catenin, up to 2-fold higher than in control, and both treatments significantly inhibited nuclear β-catenin in HCT-116 cells. Royal jelly and 10H2DA stabilized E-cadherin/β-catenin connections in the membrane area (Fig. 4, Table S4 and Fig. 5).

Promigratory and EMT marker N-cadherin significantly decreased in the HCT-116 cells after treatments (up to ~20%, and at higher dose up to ~5% of basal values in control cells). Vimentin level significantly decreased after both treatments, and 10H2DA had stronger inhibitory effect, especially at higher dose that inhibited vimentin up to ~10% of basal values. Royal jelly and 10H2DA decreased Snail dose-dependently in HCT-116 cells (Fig. 4, Table S4 and Fig. 5).

SW-480 cells showed different basal expression of antimigratory proteins: E-cadherin and nuclear β-catenin were found at lower levels, while cytoplasmic β-catenin was higher in control SW-480 cells (than in HCT-116), indicating cell stability and lower migratory potential of these cells (Table S4). Treatments increased E-cadherin in these cells, but significantly decreased the level of cytoplasmic β-catenin (Fig. 4, Table S4 and Fig. 5). According to the micrographs, nuclear β-catenin was exported from the nucleus and was found in the cytoplasm area around the nucleus (Fig. 5).

Royal jelly and 10H2DA significantly and dose-dependently inhibited all promigratory proteins and EMT markers in SW-480 cells at both concentrations. The 10H2DA was stronger in N-cadherin and Snail inhibition, but royal jelly was more effective in reduction of vimentin in SW-480 than in HCT-116 cells (Fig. 4, Table S4 and Fig. 5).

### Relative gene expression

**Fig. 6:**
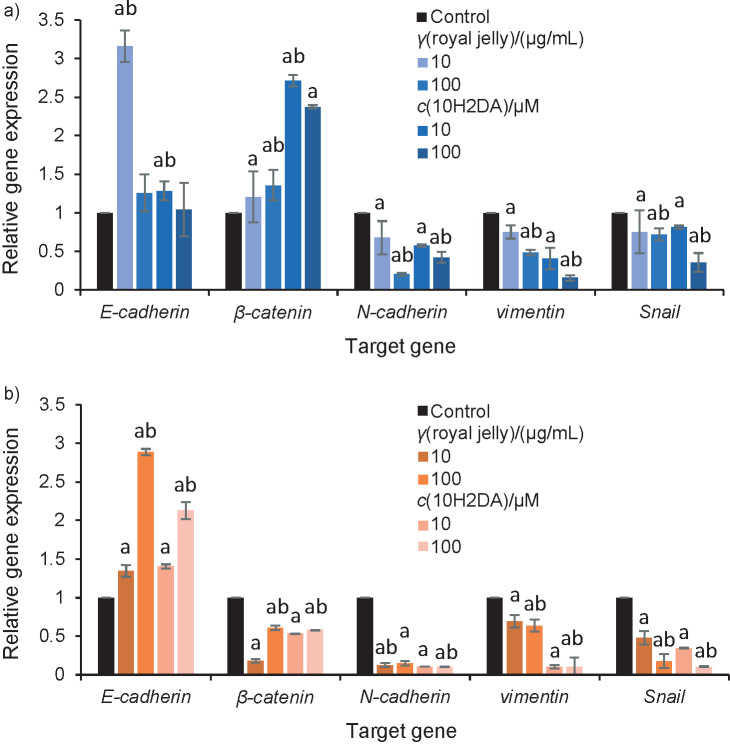
Gene expression: a) HCT-116 and b) SW-480 cells, treated with royal jelly and *trans*-10-hydroxy-2-decenoic acid (10H2DA), and presented as the fold change in mRNA expression in a target sample, normalized to a reference gene and relative to the control sample. Values of relative gene expression were calculated according to 2^-∆∆Ct^ method and *β-actin* was used as internal control gene. ^a^p<0.05 indicates a significant difference compared to control, while ^b^p<0.05 indicates a significant difference between concentrations in treated group

Increased *E-cadherin* expression in HCT-116 cells was observed after treatment with royal jelly and 10H2DA, and lower doses of both treatments caused the most significant effects. Increased *β-catenin* expression was also observed, with 10H2DA having a stronger effect on its increase. *N-cadherin, vimentin* and *Snail* were significantly decreased by both tested treatments in dose-dependent way, and 10H2DA was more effective (Fig. 6 and Table S5).

Royal jelly and 10H2DA increased dose-dependently the expression of *E-cadherin*, but significantly reduced the *β-catenin* expression in SW-480 cells. Significant and dose-dependent inhibition of promigratory/proinvasive markers *N-cadherin, vimentin* and *Snail* was also observed after both treatments, whereas 10H2DA was obviously more potent (Fig. 6 and Table S5).

Royal jelly and 10H2DA increased E-cadherin and cytoplasmic β-catenin protein and gene expression in HCT-116 cells. Obviously, these treatments caused the transport of β-catenin from the nucleus into the cytoplasm, elevating the level of available β-catenin for pairing with also increased level of E-cadherin. Thus, restoration of intercellular connections resulted in decreased EMT and migratory/invasive potential of HCT-116 cells. Furthermore, protein and gene level of all promigratory markers N-cadherin, vimentin and Snail were lowered by the treatments. It is known that acquisition of invasive phenotype and stability of E-cadherin/β-catenin complexes are in direct relation to the expression of promigratory and proinvasive markers, and upregulated E-cadherin is correlated with MMP downregulation, thus weakening the invasiveness of cells (*8*, *29*). Treatments obviously changed Wnt signalling in HCT-116 cells, further limiting migration/invasion *via* inhibition of promigratory markers that are Wnt targets.

Treatments also affected SW-480 cells, and 10H2DA was stronger in decreasing their invasiveness and collective migration. However, royal jelly and 10H2DA increased E-cadherin, lowered cytoplasmic and nuclear β-catenin and suppressed promigratory/invasive markers (also confirmed by gene expression), indicating that suppression of migration did not depend on the formation of E-cadherin/β-catenin complexes. Decrease of promigratory/invasive markers was obviously a consequence of reduced β-catenin and Snail levels in these cells. The decrease in β-catenin can be explained by the fact that our treatments as well as some other natural products are able to repress the levels of nuclear and cytosolic β-catenin in SW-480 cell line, inducing its degradation, which was already confirmed by literature data (*30*, *31*). Thus, its further accumulation in the nucleus and in the cytoplasm is prevented, disabling further progression of these cells.

Since Snail is a potent regulator of promigratory markers, EMT inducers of EMT in cancer, such as N-cadherin, vimentin and MMP-9 (*8*, *32*), it was shown that its knockdown can significantly inhibit metastasis, pointing out to Snail as an effective target for preventing it (*7*).

Generally, 10H2DA exerted more prominent effects on the tested cell lines than royal jelly, probably because this component is pure, unlike royal jelly, which is a mixture of various components (*11*, *12*, *23*).

The prominent response of HCT-116 and SW-480 cell lines was as expected, since HCT-116 cells proved to be more sensitive to natural treatments (*18*). The 10H2DA had better activity, which correlates with its antimigratory/antiinvasive effects observed in our study.

Considering scarce data on the effects of royal jelly or 10H2DA on anti- and promigratory/invasive marker expression in colorectal carcinoma cells, we found similar response of this fatty acid in human lung cancer cells, where it inhibited migration by upregulating E-cadherin and downregulating N-cadherin, vimentin and Snail (*33*). Melittin, another component of bee products, bee venom, showed the ability to downregulate *β-catenin* mRNA level, concurrently upregulating E-cadherin and lowering β-catenin in pancreatic adenocarcinoma cells (*34*), which is consistent with our results, especially in SW-480 cells. Melittin lowered mRNA and protein levels of promigratory markers in pancreatic adenocarcinoma (*34*) and liver cancer (*35*), which concords with our results, indicating that bee products are powerful in inhibiting promigratory markers in cancer.

The possible explanation for the royal jelly and 10H2DA action could be estrogenic activity of 10H2DA, considering its high affinity for binding to the estrogenic receptor β (ERβ), and activation of target genes, such as: *E-cadherin*, *N-cadherin*, *β-catenin*, *vimentin*, *Snail* (*36*). ERβ is a predominant form of estrogen receptor in CRC, and it is found in these two epithelial colorectal cancer cell lines (HCT-116 and SW-480), since it maintains the epithelial cell phenotype (*37*, *38*). Our future studies will certainly address the exploration of this estrogenic activity of royal jelly and 10H2DA related to their antimigratory effect on colorectal cancer. Also, we will focus on elucidating the differences in the types of cell migration in the CRC, as well as on the specific cellular markers involved in each type of migration/invasion.

## CONCLUSIONS

This study presents new findings and explanation of royal jelly and 10H2DA molecular mechanisms that underlie their antimigratory/antiinvasive effect on colorectal cancer cells. The 10H2DA exhibited prominent effect by attenuating promigratory/invasive markers, especially in more aggressive HCT-116 cells. This study emphasizes the significance of royal jelly and 10H2DA as potential antitumour agents, and therefore should be taken into consideration for further application in antitumour therapy.

## Figures and Tables

**Table S1:** Forward and reverse primer sequences of target genes

Gene	Forward sequence	Reverse sequence
*β-actin*	5՚-AAGCAGGAGTATGACGAGTCCG-3՚	5՚-GCCTTCATACATCTCAAGTTGG-3՚
*E-cadherin*	5'-GAACAGCACGTACACAGCCCT-3'	5'-GCAGAACTGTCCCTGTCCCAG-3'
*β-catenin*	5'-AAAATGGCAGTGCGTTTAG-3'	5'-TTTGAAGGCAGTCTGTCGTA-3'
*N-cadherin*	5'-GACGGTTCGCCATCCAGAC-3'	5'-TCGATTGGTTTGACCACGG-3'
*vimentin*	5’-GGCTCAGATTCAGGAACAGC-3’	5’-AGCCTCAGAGAGGTCAGCAA-3’
*Snail*	5’-TCAGACGAGGACAGTGGGAAAG-3’	5’-GCTTGTGGAGCAGGGACATTC-3’

**Table S2:** The effect of royal jelly and *trans*-10-hydroxy-2-decenoic acid (10H2DA) on the migration of HCT-116 and SW-480 cells. The analysis of wound space is shown as a relative level of changes of wound space compared to control cells (100%)

HCT-116	Relative wound space/%		
	*t*/h	
*γ*(royal jelly)/ (µg/mL)	0	12	24
0	100.00±0.01	(52.4±0.9)^a^	(38.4±0.8)^a^
10	100.00±0.01	(77.2±1.1)^ab^	(60.1±1.2)^ab^
100	100.00±0.01	(88.1±0.7)^ab^	(77.6±0.4)^ab^
*c*(10H2DA)/µM			
0	100.00±0.01	(52.4±0.9)^a^	(38.4±0.8)^a^
10	100.00±0.01	(80.6±1.2)^ab^	(75.0±0.8)^ab^
100	100.00±0.01	(92.5±1.0)^ab^	(80.8±0.8)^ab^
SW-480			
*γ*(royal jelly)/ (µg/mL)			
0	100.00±0.01	(64.9±1.0)^a^	(51.4±0.5)^a^
10	100.00±0.01	(87.8±1.2)^ab^	(65.1±0.5)^ab^
100	100.00±0.01	(83.4±0.6)^ab^	(76.1±1.1)^ab^
*c*(10H2DA)/µM			
0	100.00±0.01	(64.9±1.0)^a^	(51.4±0.5)^ab^
10	100.00±0.01	(8799±2.4)^ab^	(75.7±3.8)^ab^
100	100.00±0.01	(98.5±166)^b^	(85.2±2.4)^ab^

Results are presented as mean value±S.E. of two independent experiments performed in four repeats. ^a^p<0.05 is considered as statistically significant difference between treatments compared to control at the same time, and ^b^p<0.05 is considered as statistically significant difference between concentrations in a treated group

**Table S3:** Migratory and invasive potential of HCT-116 and SW-480 cells after 24 h of treatment with different concentrations of royal jelly and *trans*-10-hydroxy-2-decenoic acid (10H2DA)

Index of migration or invasion/*A*_595 nm_		
HCT-116	Migration	Invasion
Control	0.449±0.04	0.303±0.03
*γ*(royal jelly)/(µg/mL) 10	(0.34±0.03)^a^	0.27±0.0
100	(0.37±0.01)^a^	0.28±0.01
*c*(10H2DA)/µM 10	(0.35±0.01)^a^	0.25±0.01
100	(0.34±0.02)^a^	(0.22±0.01)^ab^
SW-480	Migration	Invasion
Control	0.32±0.01	0.27±0.02
*γ*(royal jelly)/(µg/mL) 10	0.31±0.01	0.23±0.01
100	(0.25±0.01)^ab^	0.22±0.02
*c*(10H2DA)/µM 10	0.32±0.01	(0.20±0.01)^a^
100	0.32±0.01	(0.19±0.00)^a^

The results are shown as the index of migratio or invasion/absorbance (*A*_595 nm_) of a mean value±S.E. from two representative experiments with four repeats. ^a^p<0.05 is considered as statistically significant difference between treatments compared to control (0 h), and ^b^p<0.05 is considered as statistically significant difference between concentrations in a treated group

**Table S4:** Protein expression of E-cadherin, cytoplasmic β-catenin, nuclear β-catenin, N-cadherin, vimentin and Snail in HCT-116 and SW-480 cells after 24 h of treatments

HCT-116	Relative fluorescence per cell				
Control	*γ*(royal jelly)/(µg/mL)		*c*(10H2DA)/µM	
0	10	100	10	100
E-cadherin	229063±14286	(315815±42224)^ab^	225086±10569	233211±12262	256808±17822
Cyt. β-catenin	233982±21882	285954±41082	300825±40804	(441501±8548)^a^	(420314±45492)^a^
Nucl. β-catenin	236446±14331	(86391±4873)^a^	(98592±14193)^a^	(111902±15083)^a^	(116397±11744)^a^
N-cadherin	1175936±78681	(235817±48593)^a^	(73712±15143)^ab^	(236668±23124)^a^	(71845±13543)^ab^
vimentin	265795±29716	(104279±8012)^a^	(112400±3427)^a^	(86382±5056)^a^	(27611±7289)^ab^
Snail	1059201±69763	(313915±12316)^a^	(64239±16773)^ab^	(126957±25256)^a^	(61528±2826)^a^
SW-480					
						E-cadherin	135741±5721	142876±6156	(276734±15416)^ab^	(187225±8306)^a^	(167475±9402)^a^
Cyt. β-catenin	586952±38904	(143203±16065)^a^	(192632±23267)^a^	(86647±9801)^a^	(147070±16421)^a^
Nucl. β-catenin	121893±19868	107706±5552	(171573±27781)^b^	120369±24178	143990±22778
N-cadherin	170722±26364	(41748±5924)^a^	(12941±1507)^a^	(17400±751)^a^	(14387±1460)^a^
Vimentin	283305±13283	(43283±3202)^a^	(43138±3482)^a^	(56968±3534)^a^	(82762.0±3469)^ab^
Snail	267145±20043	(111190±10631)^a^	(61938±8098)^ab^	(66069±8487)^a^	(53633±3912)^a^

Values are presented as a mean value±S.E. of the triplicates from two representative experiments. ^a^p<0.05 is considered as statistically significant difference between treatments compared to control (0 h), and ^b^p<0.05 is considered as statistically significant difference between concentrations in a treated group.

Cyt=cytoplasmic, Nucl=nuclear

**Table S5:** Results of gene expression are presented as the fold change in mRNA expression in a target sample, normalized to a reference gene and relative to the control sample

HCT-116	*E-cadherin*	*β-catenin*	*N-cadherin*	*vimentin*	*Snail*
Control	1	1	1	1	1
*γ*(royal jelly)/(µg/mL) 10	(3.2±0.2)^ab^	(1.2±0.3)^a^	(0.7±0.2)^a^	(0.75±0.09)^a^	(0.8±0.3)^a^
100	1.3±0.2	(1.4±0.2)^ab^	(0.20±0.02)^ab^	(0.48±0.04)^ab^	(0.72±0.08)^ab^
*c*(10H2DA)/µM 10	(1.3±0.1)^ab^	(2.71±0.08)^ab^	(0.57±0.08)^a^	(0.4±0.1)^a^	(0.81±0.02)^a^
100	1.0±0.4	(2.37±0.02)^a^	(0.42±0.07)^ab^	(0.16±0.04)^ab^	(0.4±0.1)^ab^
SW-480	*E-cadherin*	*β-catenin*	*N-cadherin*	*vimentin*	*Snail*
Control	1	1	1	1	1
*γ*(royal jelly)/(µg/mL) 10	(1.35±0.08)^a^	(0.18±0.03)^a^	(0.13±0.03)^ab^	(0.69±0.09)^a^	(0.48±0.08)^a^
100	(2.89±0.04)^ab^	(0.61±0.03)^ab^	(0.15±0.03)^a^	(0.63±0.09)^ab^	(0.18±0.08)^ab^
*c*(10H2DA)/µM 10	(1.41±0.03)^a^	(0.53±0.001)^a^	(0.11±0.001)^a^	(0.10±0.01)^a^	(0.35±0.02)^a^
100	(2.1±0.1)^ab^	(0.58±0.004)^ab^	(0.10±0.003)^ab^	(0.10±0.01)^ab^	(0.11±0.02)^ab^

Values of relative gene expression were calculated according to 2^-∆∆Ct^ method. ^a^p<0.05 indicates a significant difference compared to control, while ^b^p<0.05 indicates a significant difference between concentrations in treated group
